# Expression, purification, and characterization of biologically active full-length Mason-Pfizer monkey virus (MPMV) Pr78^Gag^

**DOI:** 10.1038/s41598-018-30142-0

**Published:** 2018-08-07

**Authors:** Fathima Nuzra Nagoor Pitchai, Lizna Ali, Vineeta Narayana Pillai, Akhil Chameettachal, Syed Salman Ashraf, Farah Mustafa, Roland Marquet, Tahir Aziz Rizvi

**Affiliations:** 1 Department of Microbiology & Immunology, College of Medicine and Health Sciences, United Arab Emirates University, Al Ain, United Arab Emirates; 2 Department of Chemistry, College of Science, United Arab Emirates University, Al Ain, United Arab Emirates; 3 Department of Biochemistry, College of Medicine and Health Sciences, United Arab Emirates University, Al Ain, United Arab Emirates; 4 Université de Strasbourg, CNRS, Architecture et Réactivité de l’ARN, UPR, 9002 Strasbourg, France

## Abstract

MPMV precursor polypeptide Pr78^Gag^ orchestrates assembly and packaging of genomic RNA (gRNA) into virus particles. Therefore, we have expressed recombinant full-length Pr78^Gag^ either with or without His_6_-tag in bacterial as well as eukaryotic cultures and purified the recombinant protein from soluble fractions of the bacterial cultures. The recombinant Pr78^Gag^ protein has the intrinsic ability to assemble *in vitro* to form virus like particles (VLPs). Consistent with this observation, the recombinant protein could form VLPs in both prokaryotes and eukaryotes. VLPs formed in eukaryotic cells by recombinant Pr78^Gag^ with or without His_6_-tag can encapsidate MPMV transfer vector RNA, suggesting that the inclusion of the His_6_-tag to the full-length Pr78^Gag^ did not interfere with its expression or biological function. This study demonstrates the expression and purification of a biologically active, recombinant Pr78^Gag^, which should pave the way to study RNA-protein interactions involved in the MPMV gRNA packaging process.

## Introduction

Retroviruses are a group of viruses that require packaging/encapsidation of their “full-length”, unspliced, single-stranded, RNA genome (gRNA) into assembling viral particles for the continuity of their life cycle. During this process, two copies of the gRNA dimerize and are preferentially packaged into the assembling virions compared to the spliced viral RNA and the large pool of cellular RNAs of the infected host cell^1–7^. Such specificity towards packaging of gRNA is a result of intricate interaction(s) between the *cis*-acting sequences on the gRNA and the *trans*-acting viral Gag protein. Retroviral *cis*-acting sequences which interact with Gag polyprotein are generally located at the 5′ end of the gRNA and have been designated as the “packaging signal” or “psi” (ψ). For almost all retroviruses, the ψ sequences required for gRNA packaging have been identified as a structurally-conserved region generally present both upstream and downstream of the major splice donor (mSD) within the 5′ untranslated region (5′ UTR) and often extending into the 5′ end of the *Gag* open reading frame (ORF)^1–6,8^.

Among the proteins implicated in selective gRNA packaging into virus particles, the nucleocapsid (NC) region of the retroviral Gag polyprotein is a primary candidate, as this highly basic protein contains Cys-His boxes that can interact with Zn^2+^ ions to facilitate protein/RNA interactions^4,9^. Mutational analysis of the NC domain of several retroviral *gag* genes has shown that it is one of the most critical proteins involved in gRNA packaging^10–14^. However, additional lines of evidence indicate that NC may not be the only determinant of specific gRNA packaging, and other Gag domains may also be involved, including matrix^15^, capsid, the p2 spacer peptide between CA and NC^16–18^, and the terminal p6 late domain^19^. Furthermore, it is thought that rather than recognizing monomeric RNA substrates, NC probably recognizes dimeric genomes, an interaction that is thought to initiate the multimerization of the Gag polyprotein on the RNA templates, eventually leading to encapsidation of the gRNA into the assembling virus particle^20–22^. Together, these observations suggests that specific selection of gRNA from cellular and spliced RNAs is a complex phenomenon that happens in the context of the whole Gag polyprotein, as has recently been shown for the human immunodeficiency virus type 1 (HIV-1)^23–26^.

**Figure 1:**
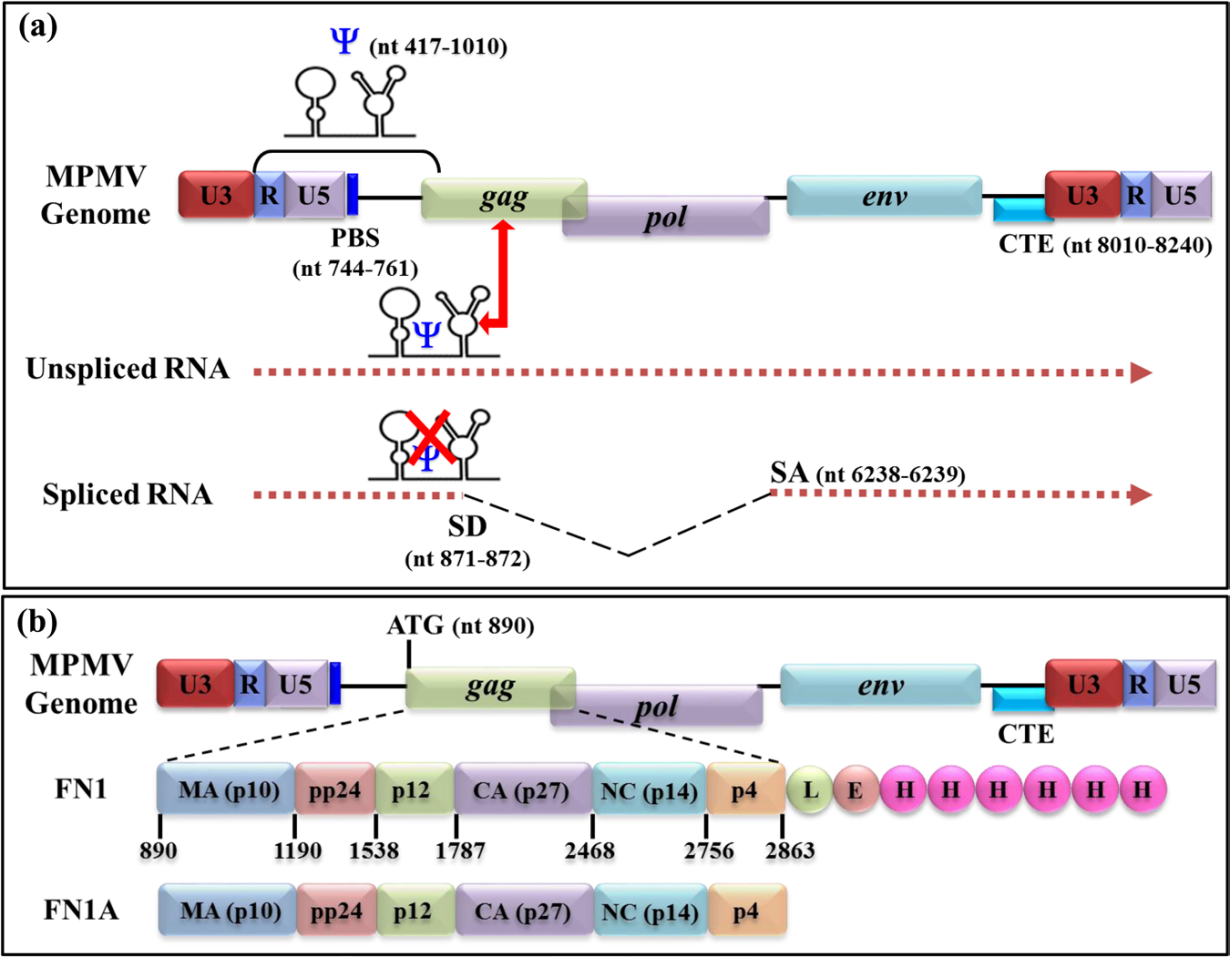
(**a**) Schematic representation of a simplistic model of retroviral genomic RNA packaging. The presence of an intact RNA secondary structure of the packaging signal (psi; ψ) facilitates genomic RNA packaging, whereas loss of its structure in the spliced RNA excludes its packaging. (**b**) Illustration of the MPMV Gag proteins expressed from the prokaryotic expression vector either with (FN1) or without (FN1A) the His_6_-tag (6xH). The amino acid residues LE were introduced upstream of the His_6_-tag into the FN1 vector during the cloning process.

Based on these observations, a simplistic model shown in Fig. 1(a) suggests that the gRNA is preferentially packaged by virtue of the presence of the *cis*-acting Gag binding site on the structured RNA packaging determinant. In the case of the spliced RNAs, part of the packaging sequence is spliced out, thereby excluding them from encapsidation into the newly formed viral particles, a process that also disrupts the structure of the packaging determinants (Fig. 1(a)). Such a model offers a possible mechanism for discriminating between spliced and unspliced viral mRNAs^1^ as has recently been shown in the case of HIV-1 where the stem loop 1 (SL1) is located in the region harboring the packaging determinants of HIV-1 and is capable of binding HIV-1 Pr55^Gag^ with high affinity^23–27^. Furthermore, the sequences downstream of this region enhance HIV-1 RNA packaging, while the sequences upstream inhibit packaging efficiency^23^. Such RNA binding studies that challenge the earlier observations where SL3 was thought to contain the primary packaging determinants^28^ have not been accomplished in most retroviruses owing to the unavailability of respective purified full-length Gag precursor proteins.

The Mason-Pfizer monkey virus (MPMV) is a non-transforming, prototypic simple, type D retrovirus, which has been shown to be involved in causing immunodeficiency in infected new-born Rhesus monkeys^29,30^. Among type D retroviruses, MPMV RNA packaging is the most thoroughly investigated^31–47^, primarily because MPMV-based vectors are considered as potential tools for delivering therapeutic genes in human gene transfer studies. MPMV-based vectors are good candidates for human gene transfer studies because: i) MPMV promoter is transcriptionally active in human cells, thereby obviating the need of replacing MPMV promoters with those of other human viral promoters, and ii) the presence of MPMV constitutive transport element (CTE) should greatly facilitate the nuclear export of the therapeutic genes for their effective expression in the target cells^34,48–50^.

A number of studies have focused on identifying the MPMV sequences (at the sequence as well as secondary RNA structure levels) responsible for gRNA packaging and dimerization^31,36–39,42,44,47^. There is now a consensus that sequences that are responsible for MPMV packaging are highly structured, bipartite in nature, and divided into two regions both upstream and downstream of the major splice donor site^31,38,39^. However, not much is known as to how Pr78^Gag^ selects gRNA. For instance, it remains largely unclear whether discrimination between gRNA and spliced RNA is mediated by the initial binding step to Pr78^Gag^, or whether other pathways such as the gRNA nuclear export and subcellular localization are also involved, as has been proposed for HIV-1^51–57^. The void in understanding selective packaging of gRNA among retroviruses is largely due to the unavailability of biologically active full-length Gag polyprotein which has been proposed to interact with the packaging sequences on the full-length, unspliced, and dimerized gRNA^23^.

The *gag* gene of MPMV encodes a polypeptide, Pr78^Gag^ that is the precursor of the viral structural proteins responsible for formation of MPMV particles. Pr78^Gag^ is proteolytically cleaved into six proteins (Fig. 1(b)): namely NH2-p10 (MA), pp24 (and its C-terminal cleaved product, referred as pp24/16), p12, p27 (CA), p14 (NC), and p4-COOH^46,58,59^. Cleavage of the polyprotein is achieved by a protease (PR) encoded for by the virally-encoded *pro* gene^58–60^. Like most retroviruses, MPMV Pr78^Gag^ assembles to form an immature capsid and expression of the *pro* gene results in the maturation of the virus particles^61^.

Since Pr78^Gag^ is a critical component of the packaging process, understanding the biochemical and biophysical properties of MPMV Pr78^Gag^ is of paramount importance to understand MPMV biology. Overexpression and purification of Pr78^Gag^ in bacteria has been reported before; however, the protein was mainly found within inclusion bodies and had to be solubilized and denatured for purification and then refolded for further analysis^62^. Furthermore, its suitability for RNA binding assays was never established. Therefore, to overcome this caveat, we have expressed large amounts of recombinant Pr78^Gag^ in soluble fractions of *Escherichia coli* (*E*. *coli*) containing a C-terminal hexa-histidine (His_6_) tag to facilitate protein purification. This was followed by immobilized metal affinity chromatography (IMAC) to purify the protein to homogeneity employing high-pressure liquid chromatography (HPLC)^19,63,64^. The availability of purified MPMV full-length Gag polyprotein should allow us to investigate how Pr78^Gag^ is involved in selectively packaging gRNA over spliced viral and cellular RNAs which will further enhance our understanding of the molecular intricacies involved in MPMV gRNA packaging, especially in delineating RNA-protein interactions that take place during MPMV replication.

## Results and Discussion

### Bacterial Expression of Recombinant MPMV Pr78^Gag^-His_6_-tag Protein

**Figure 2:**
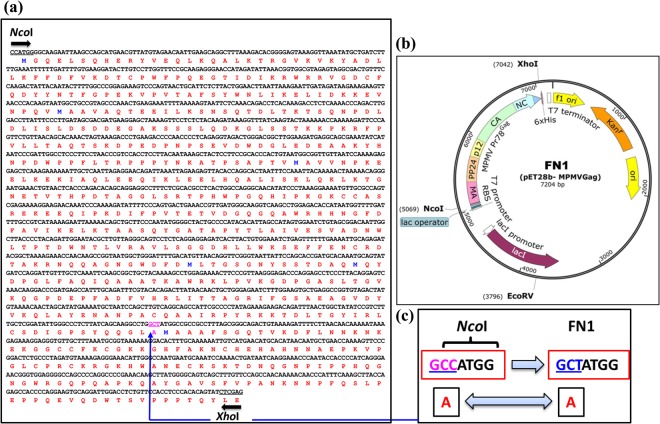
Schematic representation of the construction of the recombinant Pr78^Gag^. (**a**) Full length nucleic acid and amino acid sequence of MPMV Pr78^Gag^. (**b**) Design of the modified pET28b(+) vector expressing the full length MPMV Pr78^Gag^ (FN1) cloned into *Nco*I and *Xho*I sites and expressed from the bacteriophage T7 promoter. (**c**) A silent mutation introduced to remove an internal *Nco*I site for the ease of cloning.

In order to express full-length MPMV Pr78^Gag^, we created a recombinant bacterial expression plasmid (FN1) in a fashion that this plasmid should produce a fusion protein comprising of full-length MPMV Pr78^Gag^ and a LEHHHHHH tag at the C-terminus (Pr78^Gag^-His_6_-tag), with a predicted molecular weight of ~74101 Daltons (Fig. 1(b)). Such a T7 RNA polymerase promoter-based expression plasmid (FN1) facilitated high level expression of His_6_-tagged MPMV Pr78^Gag^ by BL21(DE3) bacterial cells when induced with IPTG because of the presence of a chromosomal copy of the T7 *RNA polymerase* gene under the dependency of the lac promoter (Fig. 2).

**Figure 3:**
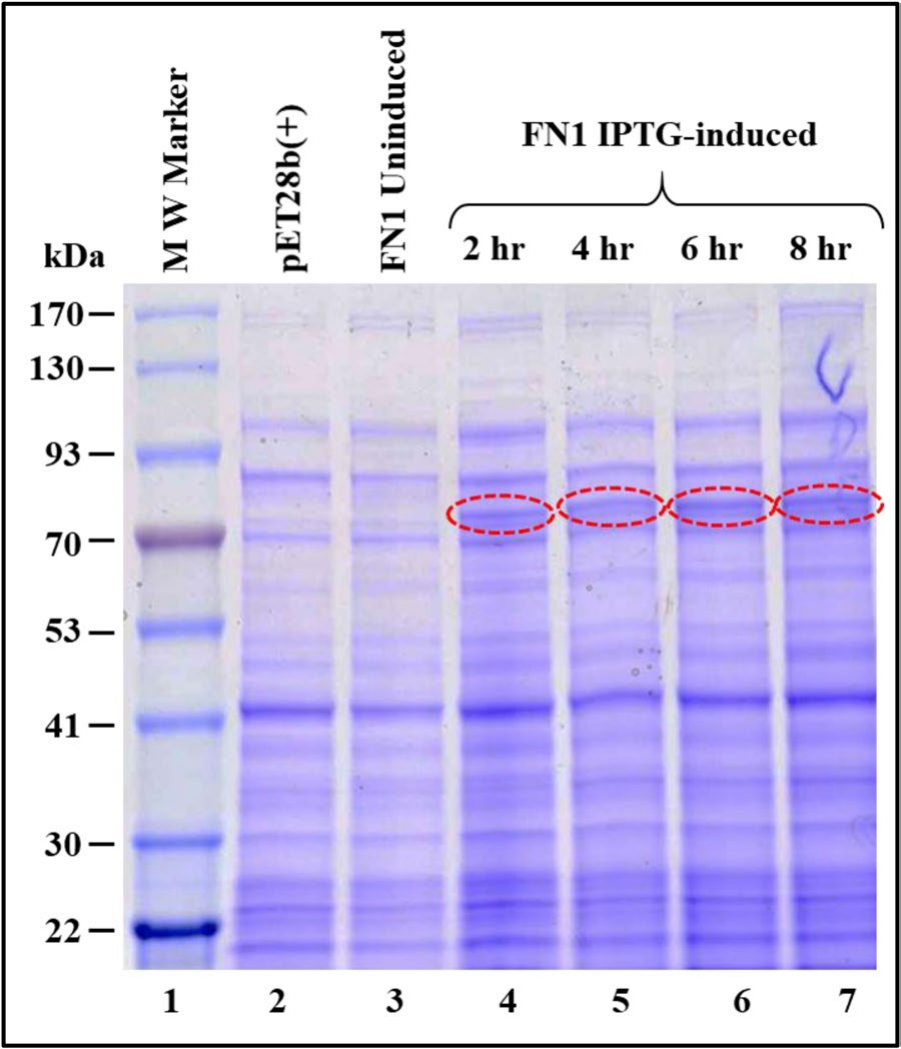
Expression of recombinant Pr78^Gag^ in *Escherichia coli* lysates. Coomassie Brilliant Blue-stained SDS-polyacrylamide gel showing expression of recombinant full-length Pr78^Gag^ prepared from total cell lysates from un-induced and IPTG-induced BL21(DE3) bacterial cells which were cultured for 0, 2, 4, 6, and 8-hours at 28 °C.

Expression of the recombinant Pr78^Gag^-His_6_-tagged protein was confirmed by growing FN1-transformed BL21(DE3) cultures at 37 °C followed by IPTG induction and were further grown at 28 °C. At 0, 2, 4, 6, and 8 hours post induction, total protein lysates were prepared from both induced as well as un-induced cultures and the expression of the recombinant MPMV Pr78^Gag^-His_6_-tag was monitored based on size on SDS-PAGE. As shown in Fig. 3, a distinct band of ~78 kDa corresponding to the expected size of the recombinant full-length Pr78^Gag^-His_6_-tag was observed in the induced cultures at 2, 4, 6, and 8 hours (lanes 4–7) but not in un-induced cultures (lane 3) or cultures containing only pET28b(+) vector without any MPMV Gag sequences (lane 2). These results reveal successful expression of the recombinant full-length MPMV Pr78^Gag^. However, we cannot be certain whether this recombinant full-length Gag protein was capable of making VLPs or was present in the soluble bacterial fraction. Klikova and colleagues have reported earlier that culturing bacteria at 37 °C post induction resulted in the confinement of MPMV Gag polyprotein in the inclusion bodies containing aberrantly assembled spiral like structures^62^. Keeping these observations in mind, the recombinant MPMV Pr78^Gag^ was expressed employing sub-optimal conditions such as low temperature in all subsequent experiments. Furthermore, although the expression of recombinant MPMV full-length Pr78^Gag^-His_6_-tag was observed between 2–8 hours post IPTG induction (Fig. 3; lanes 4–7), for subsequent experiments we chose to purify protein from cultures that were induced only for 4 hours sub-optimally at 28 °C following IPTG induction in order to avoid any possible aggregation and/or sequestration of Gag in inclusion bodies as has been reported earlier^62^.

### The Bacterially-expressed MPMV Pr78^Gag^-His_6_-tag Protein Forms VLPs

**Figure 4:**
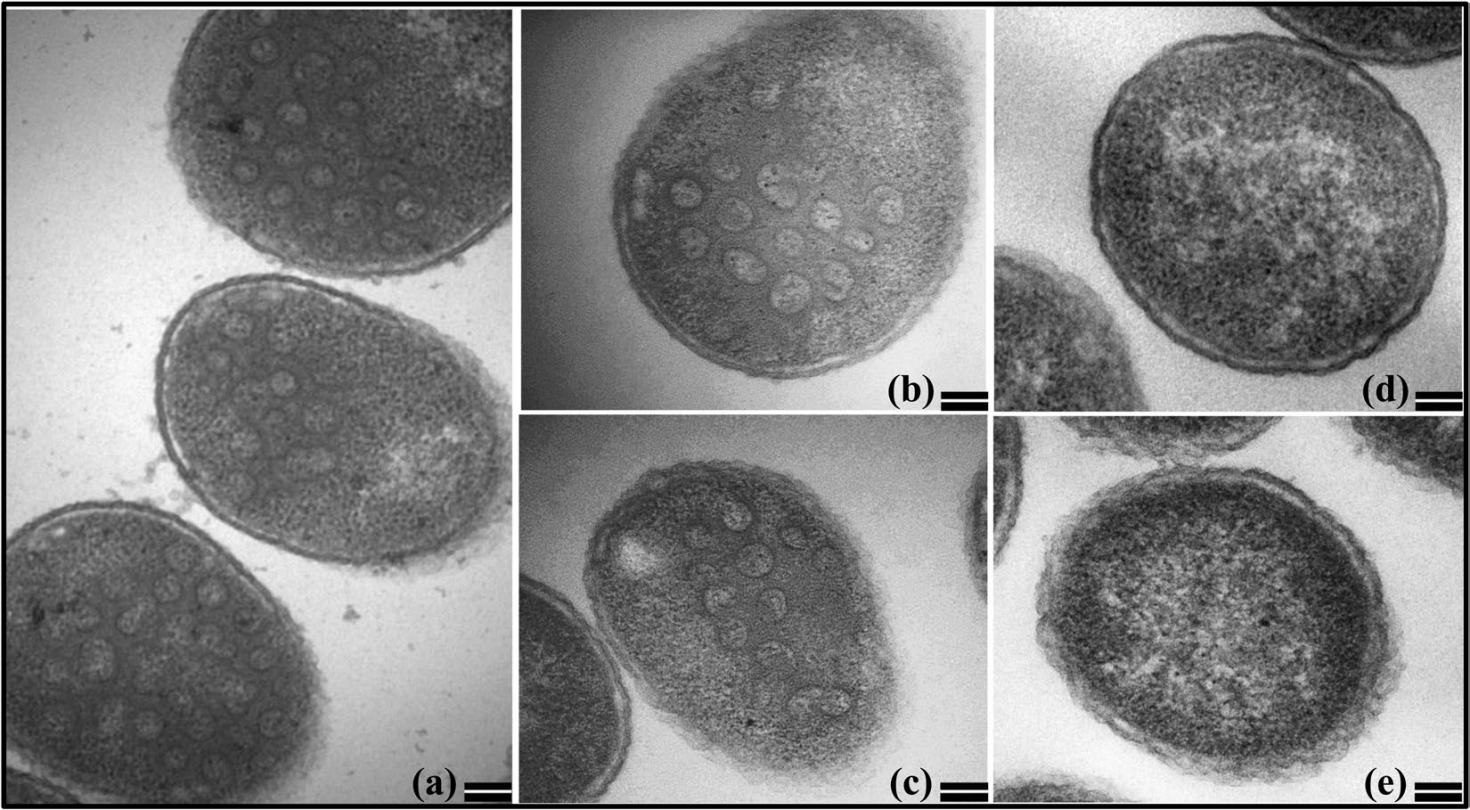
Assembly of virus like particles (VLPs) by recombinant Pr78^Gag^ in *Escherichia coli*. Transmission electron micrographs showing VLPs assembled in *E*. *coli* BL21(DE3) cells transformed with (**a**) the FN1 clone containing His_6_-tag and (**b**,**c**) with a FN1A clone without the His_6_-tag. (**d**,**e**) BL21(DE3) uninduced BL21(DE3) cells transformed with FN1 and FN1A, respectively. (Scale bar = 100 nm; 60,000X magnification).

It has previously been shown that not only MPMV Gag^62,65,66^, but other retroviral Gag proteins^67,68^ can form immature VLPs. Therefore, we tested whether the recombinant MPMV Pr78^Gag^ either with or without His_6_-tag at the C-terminus was able to assemble into VLPs within bacterial cells or the presence of His_6_-tag in any way hindered this process. Towards this end, the full-length MPMV Gag recombinant clone FN1 (with His_6_-tag) and FN1A (without His_6_-tag) were expressed in BL21(DE3) cells at 28 °C and tested for their ability to form immature VLPs using transmission electron microscopy (TEM). The ultrathin sections were negatively stained with 1% uranyl acetate and visualized. Electron micrographs of the IPTG-induced bacterial expression plasmids (FN1 and FN1A) revealed a predominant population of intra-cytoplasmic structures appearing as electron dense rings of ~55–65 nanometer (nm) in size resembling immature VLPs (Fig. 4(a–c)). This range of size (55–65 nm) of VLPs is consistent with the earlier published observations of MPMV VLPs assembled in bacteria from full-length Gag^62^ or mutated Gag^66^. No such VLP structures were observed when FN1- and FN1A-transformed bacterial cells were not induced with IPTG (Fig. 4(d,e)). Similarly, no VLP-like structures were observed when the cloning vector, pET28b(+), by itself was transformed in BL21(DE3) and induced employing similar conditions (data not shown). These results suggest that clone FN1 containing full-length MPMV Gag with a His_6_-tag at the C-terminus as well as FN1A without the His_6_-tag were able to express full-length Gag proteins capable of assembling VLPs in bacteria. Based on these results, it can be further concluded that the presence of His_6_-tag at the C-terminus does not interfere with the recombinant full-length MPMV Pr78^Gag^ expression as well as VLP formation. This observation is further strengthened by the fact that when sequences for FN1 (with His_6_-tag) and FN1A (without His_6_-tag) were subjected to online ExPASy-Compute pI/Mw tool which allows the computation of the theoretical isoelectric point (pI) of proteins, it predicted minimal difference (0.15) in these proteins (FN1-with His_6_-tag: pI: 7.07 *versus* FN1A-without His_6_-tag: pI: 7.22).

### MPMV Pr78^Gag^-His6-tag Protein is Expressed in the Soluble Fraction in Bacteria

**Figure 5:**
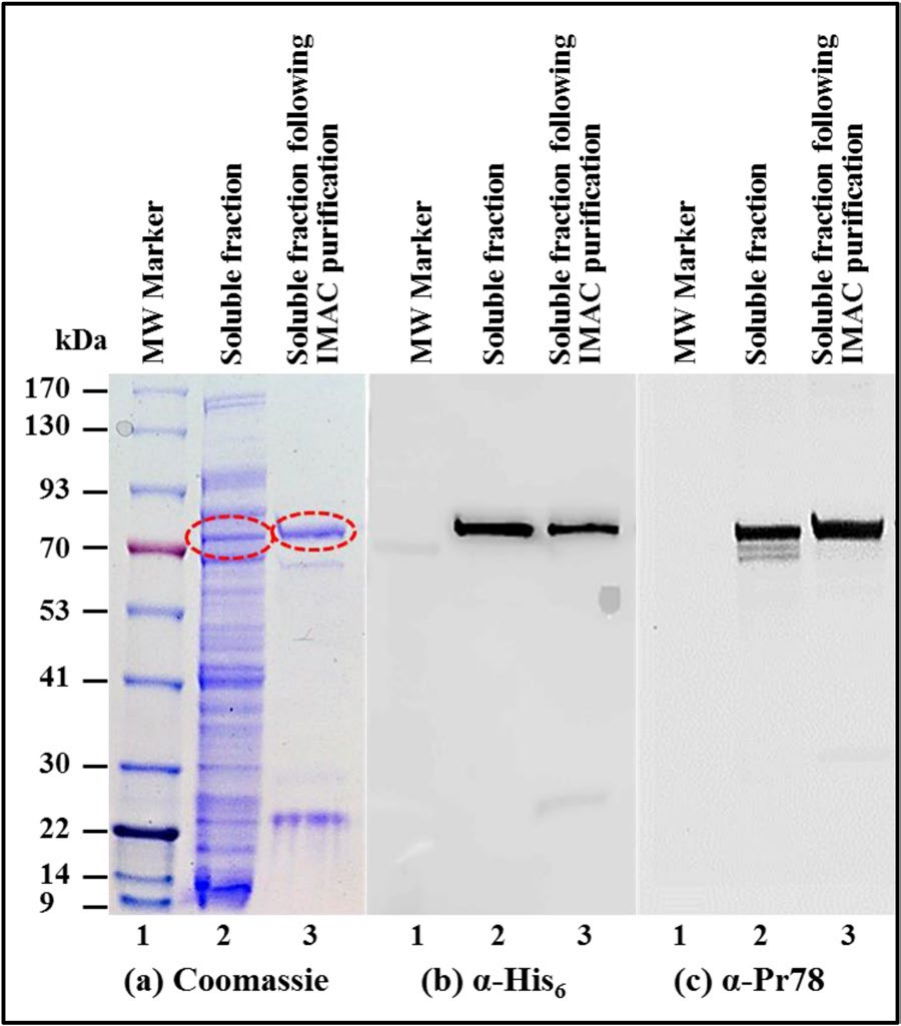
Expression of recombinant Pr78^Gag^ in the soluble fraction of *E*. *coli*. (**a**) Coomassie Brilliant Blue-stained SDS-polyacrylamide gel with lysates from the soluble fraction of bacteria transformed with FN1 expressing recombinant full-length MPMV Pr78^Gag^-His_6_-tag fusion protein (lane 2), followed by IMAC purification (lane 3). The same lysates were analyzed with (**b**) a monoclonal anti-His_6_ monoclonal antibody, and (**c**) an anti-Pr78^Gag^ polyserum, respectively.

In order to determine whether the recombinant MPMV Pr78^Gag^-His_6_-tag protein was present in the soluble bacterial fraction, expression of the full-length MPMV Gag clone in FN1 was induced with IPTG at 28 °C for 4 hours and lysed as described in Material and Methods. Insoluble material (containing cell debris and inclusion bodies, if any) was removed by centrifugation and the soluble fraction from different cultures was either stored at −80 °C or immediately monitored for the expression of the recombinant MPMV Pr78^Gag^-His_6_-tag protein by SDS-PAGE and immunoblotting. Staining with Coomassie Brilliant Blue revealed presence of a distinct band of ~78 kDa corresponding to the expected size of recombinant MPMV Pr78^Gag^-His_6_-tag protein (Fig. 5(a); lane 2). The identity and recombinant nature (Pr78^Gag^-His_6_-tag fusion protein) of this band was established by immunoblotting using HRP-conjugated α-His_6_ monoclonal antibody (Fig. 5(b); lane 2) as well as α-MPMV Pr78 polyserum (Fig. 5(c); lane 2). These results show that under the conditions used by us, the recombinant Pr78^Gag^-His_6_-tag protein was expressed primarily in the soluble fraction in contrast to the inclusion bodies, as reported earlier^62^.

### Further Purification of the Soluble Fraction Containing Pr78^Gag^-His_6_-tag Fusion Protein by Immobilized Metal Affinity Chromatography (IMAC)

Having established that the expressed recombinant Pr78^Gag^-His_6_-tag protein was soluble, we wanted to further increase the purity of our protein since two additional faint bands could be observed underneath the primary protein band when detected by the α-MPMV Pr78 polyserum, but not by α-His_6_ antibody which could be degraded products (Fig. 5(b) *versus* Fig. 5(c)). Thus, the purified bacterial lysate (containing soluble fraction of recombinant Pr78^Gag^-His_6_-tag protein) was further clarified employing IMAC, as described in Materials and Methods. The buffering conditions used in these protocols (non-denaturing conditions and especially the presence of 1.0 M NaCl), not only allowed the protein to bind to the column, but also avoided protein aggregation and precipitation. The IMAC-purified protein was then monitored for purity of the recombinant MPMV Pr78^Gag^-His_6_-tag protein by SDS-PAGE and immunoblotting. Coomassie Brilliant Blue stain of the SDS-PAGE demonstrated that most bacterial proteins that were present in the soluble fraction prior to IMAC purification were eliminated following IMAC purification (compare lane 2 with lane 3 in Fig. 5(a)). Immunoblotting of IMAC-purified protein with HRP-conjugated anti-His_6_ monoclonal antibody (Fig. 5(b); lane 3) and anti-MPMV Pr78 polyserum (Fig. 5(c); lane 3) further confirmed the purity of the protein, as observed by the disappearance of the additional faint bands seen earlier (compare lane 2 with lane 3 in Fig. 5(c)). These results confirm that MPMV full-length Gag is truly fused with the His_6_-tag, allowing its binding to the HisTRAP^TM^ column and subsequent elution in the purified form, further authenticating the recombinant nature of MPMV Pr78^Gag^- His_6_-tag fusion protein (compare Fig. 5(b); lane 3 with Fig. 5(c); lane 3).

### Concentration and further Purification of the IMAC-purified Pr78^Gag^-His_6_-tag Protein by Gel Filtration Chromatography

**Figure 6:**
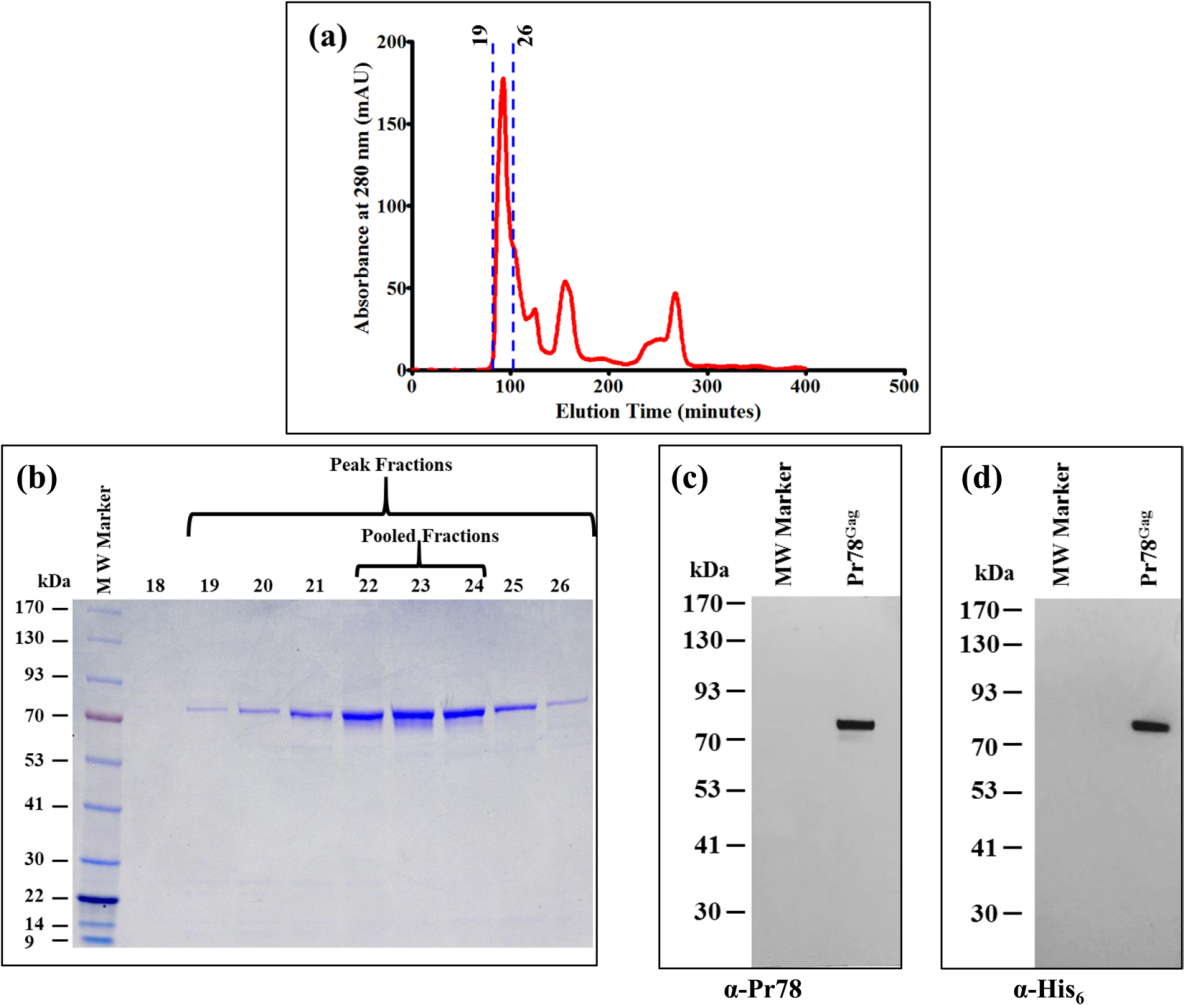
Fractionation of IMAC-purified recombinant Pr78^Gag^ protein by size exclusion chromatography. (**a**) Absorbance *versus* elution time chromatogram plotted from the data obtained from a Superdex 200 column showing peak fractions with maximum absorbance containing purified recombinant full-length MPMV Pr78^Gag^-His_6_-tag fusion protein expressed from FN1. (**b**) Coomassie Brilliant Blue-stained SDS-polyacrylamide gel showing the resolution of purified recombinant full-length MPMV Pr78^Gag^-His_6_-tag fusion protein expressed from FN1 in fractions 18–26. (**c**) Western blot analysis of pooled peak fractions (22–24) of purified recombinant full-length MPMV Pr78^Gag^-His_6_-tag fusion protein analyzed with anti-Pr78^Gag^ polyserum, and (**d**) anti-6x-His monoclonal antibody, respectively.

Following IMAC purification, the bacterially-expressed full-length Pr78^Gag^-His_6_-tag protein was concentrated and further purified by gel filtration/size exclusion chromatography under non-denaturing conditions. As in the case of IMAC purification, non-denaturing conditions (especially the presence of 1.0 M NaCl) were employed to prevent protein aggregation and precipitation. Based on a sharp absorbance peak at 280 nm, fractions of 500 µl each were collected over several hours (Fig. 6(a)). Additionally, two other smaller peaks were also noticed that represented the degradation products or unidentified proteins of much lower molecular weight compared to Pr78^Gag^-His_6_-tag protein, as established by SDS-PAGE analysis (data not shown), and therefore were eliminated for any further downstream applications.

Protein fractions representing the sharp peak (fractions 19–26) were further analyzed by separation on SDS-PAGE. As shown in Fig. 6(b), fractions collected from the MPMV Pr78^Gag^-His_6_-tag peak were characteristically pure, with varying amounts of protein. Fractions representing the highest amount of pure protein (peaks 22–24) were pooled and once again concentrated using Ultra 15 (30,000 molecular weight cut-off membrane) concentrators. To establish the purity of recombinant full-length MPMV Pr78^Gag^-His_6_-tag Gag fusion protein, we measured the A260/A280 ratio by spectrophotometry. Our spectrophotometric analysis revealed the A260/A280 ratio to be 0.61, indicating that our recombinant full-length MPMV Pr78^Gag^-His_6_-tag fusion protein’s purity was greater than 95%. The concentrated protein was further analyzed by immunoblotting using anti-MPMV Pr78 polyserum and HRP-conjugated anti-His_6_ monoclonal antibody. Figure 6(c,d), in close corroboration with the SDS-PAGE analysis (Fig. 6(b)), clearly demonstrate that the pooled protein fractions contained pure MPMV Pr78^Gag^-His_6_-tag fusion protein. The protein yield, following IMAC purification and gel filtration/size exclusion chromatography, was estimated to be 3.8 mg and 0.23 mg per liter, respectively.

### The Recombinant Pr78^Gag^-His_6_-tag Protein can Assemble *in Vitro* to Form VLPs

The recombinant full-length Gag proteins from HIV-1 and feline immunodeficiency virus (FIV) have been shown to assemble *in vitro* to form VLPs^19,64,69,70^. Therefore, we tested the ability of our purified recombinant full-length MPMV Pr78^Gag^-His_6_-tag fusion protein expressed in bacteria to assemble *in vitro* to form VLPs. Since the presence of nucleic acids along with purified Gag protein has been shown to be a prerequisite for VLP formation^19,64,69,70^, therefore, purified recombinant Pr78^Gag^-His_6_-tag fusion protein was mixed with yeast tRNA, as described in Materials and Methods (in an appropriate buffer to avoid formation of protein aggregates). This mixture was then dialyzed against a buffer that was of low salt concentration compared to the buffer in which the protein-RNA mixture was prepared. As an appropriate negative control, only yeast tRNA was also dialyzed without any protein in the same buffer and manner as the protein-RNA mixture. Following dialysis, the protein-RNA mixture as well as the control tRNA suspension (without any protein) was recovered from the cassette and concentrated after which ~1/25^th^ of the concentrate was spotted onto carbon coated grids, dried, and stained for observation under an electron microscope.

**Figure 7:**
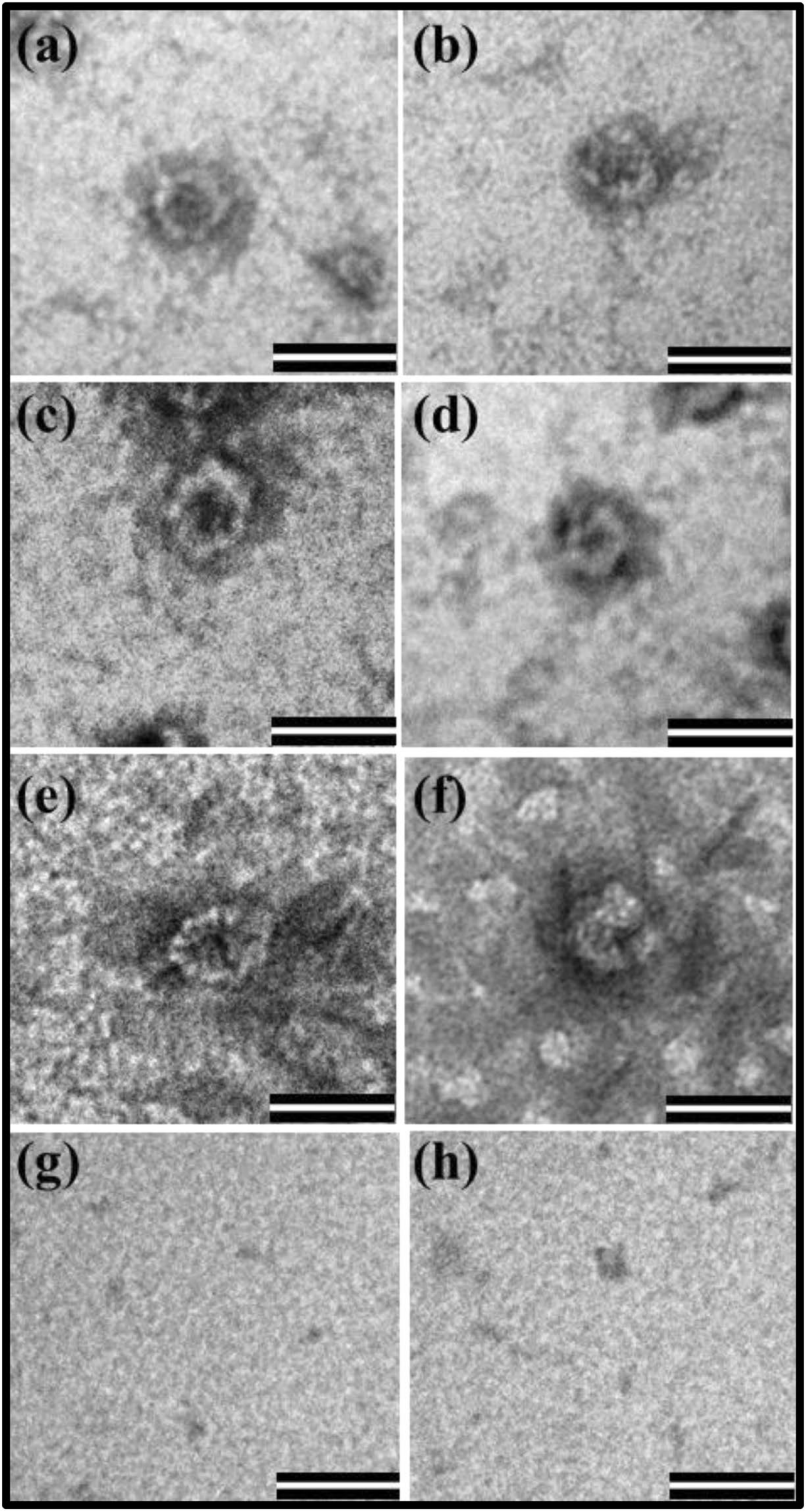
*In vitro* assembly by purified recombinant Pr78^Gag^ protein to form virus like particles (VLPs). (**a–f**) Transmission electron micrographs showing *in vitro*-assembled virus like particles from purified recombinant full-length MPMV Pr78^Gag^-His_6_-tag fusion protein expressed from FN1 in the presence of yeast tRNA. (**g**,**h**) Electron micrographs of negative controls comprising of assembly buffer and yeast tRNA only without any protein. Scale bar = 50 nm; 135,000X magnification.

Analysis of various electron micrographs taken from different fields revealed the assembly of VLPs in the form of compact electron-dense rings of approximately 30–35 nm in size resembling immature VLPs (Fig. 7(a–f)). The size of these *in vitro* assembled VLPs corroborated well with the earlier observations which have reported a similar size (~20–30 nm) obtained following *in vitro* assembly using purified Gag as opposed to the larger size of full-length Gag particles (~55–65 nm) produced *in vivo* in eukaryotic cells^19,64,69,70^. These VLPs were assembled efficiently despite the fact that the purified protein was frozen and thawed which suggests that the purified protein remained biologically active following freeze-thaw cycle. In contrast, yeast tRNA alone, without any purified MPMV full-length Gag, did not show any VLP-like structure (Fig. 7(g,h)). These results suggest that MPMV recombinant full-length Gag His_6_-tag fusion protein has the inherent property of forming VLPs by virtue of its intrinsic multimerizing ability, as has been reported previously in the case of HIV-1^19,64,69–71^.

### Recombinant Pr78^Gag^-His_6_-tag Protein Expressed in Eukaryotic Cells can form VLPs Capable of Packaging Unspliced Transfer Vector RNA

**Figure 8:**
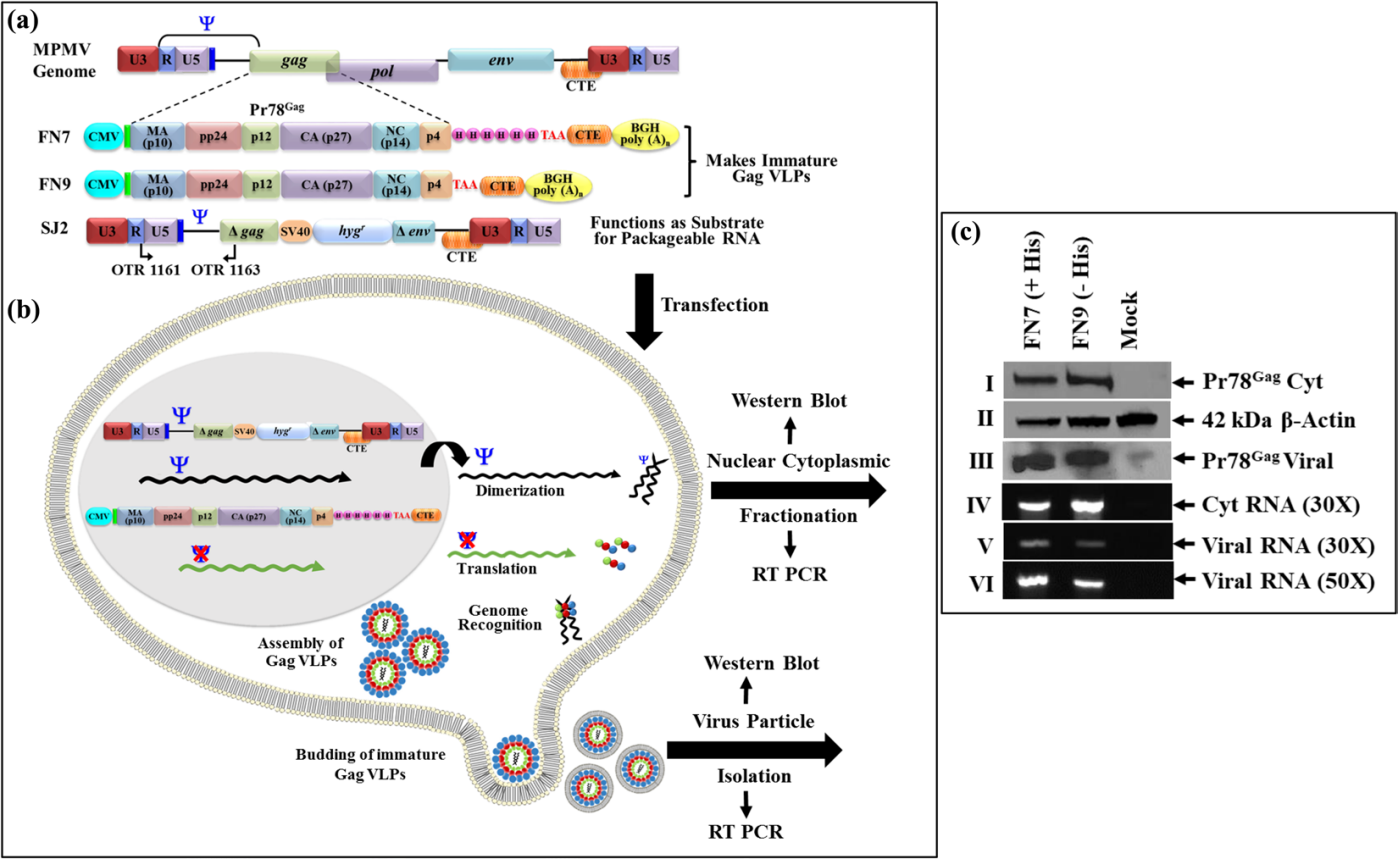
Two-plasmid genetic complementation assay to test the ability of MPMV virus like particles (VLPs) to package transfer vector viral RNA following Pr78^Gag-^His_6_-tag expression in eukaryotic cells. (**a**) Graphical representation of MPMV full-length Gag expression plasmids with and without His_6_-tag (FN7 and FN9 respectively) and MPMV transfer vector, SJ2^38^ which provides the substrate for packageable RNA. (**b**) Design and rationale of the MPMV 2-plasmid genetic complementation assay. VLPs produced by the eukaryotic Gag-expression plasmids (FN7 and FN9) should allow packaging of the transfer vector RNA expressed from SJ2 due to the presence of the packaging signal (Ψ) on its RNA. The 293T cells were co-transfected with the either of the two Gag-expression plasmids along with SJ2 and fractionated into nuclear and cytoplasmic fractions. The cytoplasmic fractions were analyzed for transfer vector (SJ2) RNA expression, while the virus particles were tested for their ability to package SJ2 RNA using RT- PCR. (**c**) MPMV full-length Gag expression plasmids with (FN7) and without His_6_-tag (FN9) were transfected into 293T cells along with SJ2 and western blots were performed on cell lysates to detect Gag proteins using anti-Pr78^Gag^ polyserum (panel I) and β-actin proteins using a monoclonal antibody as a control (panel II). Western blots on ultracentrifuged virus particles using anti-Pr78^Gag^ polyserum (panel III). RT-PCR using MPMV transfer vector (SJ2)-specific primers (OTR1161 and OTR1163) amplifying a 530 bp fragment from the cDNAs prepared from the cytoplasmic (panel IV) and virion RNAs amplified for 30 (panel V) and 50 (panel VI) cycles, respectively. Experiments were conducted multiple times with reproducible results and representative blots are shown. Blots/gels have been cropped to show the relevant parts only and full images are provided in Supplementary Data.

Finally, in order to ensure that MPMV full-length Pr78^Gag^-His_6_-tag is able to encapsidate transfer vector RNA in eukaryotic cells, we created two full-length MPMV Gag eukaryotic expression plasmids, with or without the His_6_-tag (FN7 and FN9, respectively (Fig. 8(a)). To ensure proper export of the MPMV Pr78^Gag^-His_6_-tag mRNA out of the nucleus, the MPMV CTE was inserted immediately downstream of the MPMV Gag stop codon (Fig. 8(a)). The ability of these full-length Gag expression plasmids to encapsidate MPMV RNA was tested by employing a two-plasmid genetic complementation assay (Fig. 8(b)). In this assay, either FN7 or FN9 were co-transfected with the MPMV transfer vector, SJ2, to test their ability to package the MPMV transfer vector RNA expressed from SJ2^38^. To monitor transient transfection efficiencies, a secreted alkaline phosphatase (SEAP) expression plasmid (pSEAP) was also included in the transfection DNA cocktail.

As can be seen in Fig. 8, the transfected 293T cells revealed successful expression of the full-length MPMV Pr78^Gag^ proteins using anti-Pr78^Gag^ polyserum and anti-β-Actin monoclonal antibody as a control (Fig. 8(c); panels I and II, respectively). Western blot analysis of virus particles isolated from transfected culture supernatants further confirmed VLP production by both the His(+) and His(−) Gag-expression plasmids, FN7 and FN9, respectively (Fig. 8(c); panel III). These results clearly demonstrate that the presence of His_6_-tag at the C-terminus of MPMV full-length Pr78^Gag^ did not interfere with the expression of recombinant full-length MPMV Pr78^Gag^-His_6_-tag fusion protein or its ability to form Gag VLPs in 293T cells.

Next, we tested the ability of VLPs produced by the recombinant MPMV full-length Pr78^Gag^ in eukaryotic cells to package MPMV transfer vector RNA. Towards this end, RNA was extracted from the cytoplasmic fractions as well as the pelleted viral particles and DNase-treated to deplete any contaminating plasmid DNA from the transfected cultures. PCR conducted on the DNased-RNAs using transfer vector RNA-specific primers (OTR 1161 and OTR 1163) confirmed the absence of any contaminating plasmid DNA in the RNA preparations (see Supplementary Data) and this was followed by their conversion into cDNAs. We also ensured that our fractionation technique was not compromised and there was no leakage of RNA from the nucleus to the cytoplasm by testing for the absence of unspliced actin mRNA in our cytoplasmic fractions during this process (data not shown), as described by our group earlier^38,39,72–74^. RT-PCR amplification of transfer vector (SJ2)-specific RNA revealed its successful nuclear transport and expression in the cytoplasm (Fig. 8(c); panel IV). Amplification of the SJ2-specific cDNAs isolated from Gag VLPs formed by either the MPMV Pr78^Gag^-His_6_-tag fusion protein (FN7) or without His_6_-tag (FN9) revealed that transfer vector RNA was efficiently packaged by both types of VLPs (Fig. 8(c); panels V and VI). Interestingly, the VLPs formed by the C-terminally His_6_-tagged version of the Gag protein seemed slightly more efficient in encapsidating the transfer vector RNA than its untagged version (Fig. 8(c); panels V and VI). Since the Gag NC is thought to be the main domain involved in the interaction with the genomic RNA using its positively-charged zinc finger domains, addition of the His_6_-tag may have increased the basic nature of the polyprotein and stabilized its interaction with the RNA further. Therefore, the slight increase in packaging could be attributed to the increased positive charge added to the full-length MPMV Pr78^Gag^ due to the presence of positively charged His_6_-tag, as has recently been suggested for HIV-1 Pr55^Gag^- nucleic acid interaction *in vitro*^63^.

## Conclusions

Together, this study reveals that the recombinant MPMV Pr78^Gag^- His_6_-tag fusion protein has the ability to form VLPs in both prokaryotic and eukaryotic cells. Furthermore, Gag VLPs formed in the eukaryotic cells have the capability of encapsidating transfer vector RNA, one of the important biological roles of Gag. The availability of relatively large amounts of bacterially expressed, biologically active, full-length MPMV Pr78^Gag^ protein, as described here, offers a range of opportunities to study RNA-protein interactions that take place during MPMV gRNA packaging and viral assembly. One of the interesting research areas that can now be explored are co-crystallization experiments with Pr78^Gag^ and viral genomic RNA. Having crystal structures of viral RNA-Gag complexes would allow for confirmation of proposed MPMV RNA secondary and tertiary structures, as well as proposed Gag-RNA interactions. Such refinement in the understanding of these crucial steps in retroviral life cycle is essential if MPMV-based vectors are to be used for gene therapy.

## Methods

### Nucleotide numbering system

Nucleotide numbers in this study refers to the MPMV genome with the Genbank accession number M12349^46^.

### Construction of Prokaryotic MPMV Gag Expression Plasmids

MPMV *gag* gene (Pr78^Gag^) harboring sequences spanning nucleotides 890 to 2863 was commercially synthesized as a double-stranded DNA fragment (Macrogen, South Korea). Two restriction sites, *Nco*I and *Xho*I, were incorporated at either ends of the gene to facilitate cloning into the prokaryotic expression vector pET28b(+) (Fig. 2(a)). Use of the *NcoI* site pET28b(+) vector kept the *gag* open reading frame (ORF) intact and allowed the introduction of a hexa-histidine (His_6_-tag) at the C-terminus of the Pr78^Gag^, allowing purification of the expressed recombinant Gag polyprotein using IMAC^63,64^. Since the MPMV *gag* gene contained an additional internal *NcoI* site, a silent mutation was introduced into the internal *NcoI* site at the gene synthesis step at nt 2462, changing CCATGG to CTATGG, which resulted in the loss of the *NcoI* site; while maintaining the same amino acid (Fig. 2(a and c)). The resultant clone FN1 was sequenced to ensure that the MPMV *gag* gene did not contain any point mutations (Fig. 2(b)). Such an expression plasmid should produce a recombinant fusion protein comprising of full-length MPMV Pr78^Gag^ and a LEHHHHHH tag at the C-terminus (Pr78^Gag^-His_6_-tagged), with a predicted molecular weight of 74101 daltons. Employing a similar strategy, another bacterial expression plasmid (FN1A) was constructed expressing full-length MPMV Pr78^Gag^ without the His_6_-tag (Fig. 1(b)).

### Construction of Eukaryotic MPMV Gag Expression Plasmids

The full length Pr78^Gag^ sequences both with and without the His_6_-tag were also cloned into the eukaryotic expression vector pcDNA3. Towards this end, the FN1 plasmid was used as a template for amplification of *gag* gene sequences using the forward primer, OTR1330 and the reverse primers, OTR1331 or OTR1320. OTR1330 (5′ CCG *CTC GAG* GCC GCC ACC ATG GGG CAA GAA TTA AGC CAG G 3′) introduced an *Xho*I restriction site (italicized) followed by Kozak sequence (underlined) at the 5′ end of the *gag* gene to enhance gene expression. OTR1331 (5′ CAA GGT GGA GGG TGT GTC ATA GTG GTG GTG GTG GTG GTG ATT *GAG CTC* GCC 3′) on the other hand, created a His_6_ tag (underlined) just upstream of the *gag* stop codon, followed by the *Xho*I restriction site (italicized). To create a clone without His_6_ tag, the reverse primer OTR1320 (5′ CCG *CTC GAG* TTA ATA CTG TGT GGG AG 3′) was used, which did not contain the His_6_ tag sequences but did contain an *Xho*I restriction site (italicized). Polymerase chain reaction (PCR) was performed using an initial denaturation at 98 °C for 30 seconds, then 15 cycles of denaturation at 98 °C for 10 seconds, primer annealing at 62 °C for 30 seconds, followed by primer extension at 72 °C for 30 seconds. A final extension step at 72 °C for 10 minutes was also included. The PCR-amplified products were cleaved with *Xho*I endonuclease and cloned into pCDNA3 which had previously been cleaved with the same restriction endonuclease to create clones FN3 and FN5, with and without His_6_-tag, respectively. Finally, to facilitate efficient nuclear export of *gag* mRNA, a PCR-amplified fragment containing the MPMV CTE^34^ with flanking *Xba*I sites was cloned into FN3 and FN5 which had already been cleaved with *Xba*I, resulting in clones FN7 and FN9, respectively (Fig. 8(a)). All clones were confirmed by sequencing.

### Bacterial Strains and Media

During the course of cloning, all transformations were performed in the DH5α strain of *E*. *coli* using standard heat shock protocol and allowed to grow on Luria-Bertani (LB) agar plates in the presence of appropriate antibiotics (kanamycin at 50 µg/ml and ampicillin at 100 µg/ml), depending on the clones. To monitor the recombinant protein expression in bacteria, the prokaryotic expression clones were transformed into T7 Express (New England Bio Labs, USA), a BL21(DE3) strain of *E*. *coli*. For bacterial expression studies, cells were cultured in LB medium [1% (w/v) peptone, 0.5% (w/v) yeast extract, and 0.5% NaCl] in the presence of kanamycin (50 µg/ml) antibiotic.

### Large Scale Expression of Recombinant Pr78^Gag^-His_6_-tagged Protein

Large scale recombinant Pr78^Gag^-His_6_-tagged protein expression was performed by inoculating a single colony into 25 ml LB media containing kanamycin antibiotic (50 µg/ml), and cultured at 37 °C, overnight with shaking at 200 rounds per minute (rpm). The overnight culture was used to inoculate 500 ml LB media containing kanamycin (50 µg/ml) in a 2-liter baffled Erlenmeyer flask. The culture was grown at 28 °C until an OD_600_ nm of approximately 0.6 was reached. Protein expression was induced with the addition of 0.4 mM IPTG, and the cells were grown for a further 4 h at 28 °C. Cells were harvested by centrifugation (6,300 *g*) at 4 °C for 15 minutes and cell pellets were stored frozen at −80 °C.

### IMAC Protein Purification and Size Exclusion Chromatography

Recombinant Pr78^Gag^-His_6_-tag protein was purified, as has been described previously^19,63,64^. Briefly, IPTG-induced bacterial pellets were lysed in cold CelLytic B (Sigma-Aldrich) supplemented with 500 units of Benzonase (Merck), 0.2 mg/ml lysozyme (Sigma-Aldrich) and 1X concentration of EDTA-free protease inhibitor tablet (Roche). The soluble fraction was collected after centrifugation (48,000 *g* for 1 hour) at 4 °C and the supernatant was diluted with 4X binding buffer (0.2 M Tris-HCl (pH 8.0), 4.0 M NaCl, 40 mM ß-mercaptoethanol, 10 mM dithiothreitol, 100 mM imidazole, 0.4% (w/v) Tween-20) to a final concentration of 1X. The lysate was then filtered through a 0.4 µm Polyethersulfone (PES) syringe filter and loaded onto a 5 ml HisTRAP^TM^ FF (fast flow) cartridge (GE Healthcare) that had been equilibrated with equilibration buffer (50 mM Tris-HCl (pH 8.0), 1.0 M NaCl, 10 mM ßmercaptoethanol, 2.5 mM dithiothreitol, 25 mM imidazole, 0.1% (w/v) Tween-20, and 10% (v/v) glycerol). After loading the filtrate, the column was washed with the same equilibration buffer except it contained 50 mM imidazole and the bound proteins were eluted with equilibration buffer containing 250 mM imidazole.

Following HisTRAP^TM^ column elution, Pr78^Gag^-His_6_-tag protein was concentrated using Amicon^®^ Ultra 15 (30,000 molecular weight cut-off membrane) and was further fractionated by gel filtration/size exclusion chromatography using a Superdex 200 10/300 GL column (GE Healthcare) previously equilibrated with 50 mM Tris-HCl (pH 8.0) and 1.0 M NaCl. Following SDS-PAGE analysis, peak fractions containing Pr78^Gag^-His_6_-tag protein were pooled and stored for long term usage in 2 µg/µl aliquots at −80 °C.

### SDS-polyacrylamide Gel Electrophoresis and Western Blotting

Expression and purification of recombinant Pr78^Gag^-His_6_-tag protein was monitored by SDS-PAGE and western blotting. Briefly, protein samples were mixed with 6X SDS dye, boiled for 5 minutes before loading onto a 4–12% ExpressPlus^TM^ PAGE gel (GenScript), electrophoresed under reducing conditions using MOPS buffer (GenScript), followed by their staining with Coomassie Brilliant Blue. Recombinant Pr78^Gag^ expression and purification was further monitored by transferring non-stained gels onto a nitrocellulose membrane and blotting with anti-rabbit MPMV Gag/Pol Pr78 polyserum (kindly provided by Dr. Eric Hunter, Emory University, Atlanta, GA) and with an anti-His_6_ monoclonal antibody-HRP conjugate (Sigma-Aldrich).

### Eukaryotic Expression of Recombinant Pr78^Gag^-His_6_-tag Protein

Transient transfections of the expression vectors (4 micrograms (µg) of full-length Gag eukaryotic expression plasmids (FN7 and FN9) along with 2 µg of MPMV-based transfer vector SJ2^38^ were carried out in HEK 293T cells in triplicates using a calcium phosphate kit (Invitrogen) following manufacturer’s recommendations. The resulting supernatants from the transfected cultures containing virus particles were subjected to low-speed centrifugation (bench-top centrifuge, 3,700 *g* for 10 minutes) to clear cellular debris. Next, supernatants were filtered using 0.2 μm surfactant free cellulose acetate (SFCA) syringe filters and subjected to ultracentrifugation at 70,000 *g* to pellet virus like particles (VLPs) using a 20% (w/v) sucrose cushion. The pelleted VLPs were resuspended in TN buffer (20 mM Tris-HCl (pH 7.4), 150 mM NaCl) and processed for RNA extraction and western blotting. RNA isolation was performed using TRIzol®.

### Reverse Transcriptase Polymerase Chain Reaction (RT-PCR)

Viral RNA preparations were treated with Turbo DNase (Invitrogen) and amplified using transfer vector (SJ2)-specific primers, OTR1161 (5′ GAT CAG AAC ACT GTC TTG TC 3′) and OTR1163 (5′ CTT TCT TAT CTA TCA ATT CTT TAA 3′), to ensure that the RNA preparations were not contaminated with any plasmid DNA that may have been carried over from the transfected cultures. Next, the DNAsed-RNAs were converted into cDNAs using random hexamers (5′ NNNNNN 3′) and MMLV reverse transcriptase (Promega, USA) as described previously^75,76^. cDNAs were amplified using the same vector-specific primers (OTR1161 and OTR1163) to monitor the ability of Pr78^Gag^ VLPs to package transfer vector (SJ2) RNA, as described previously^38^.

### VLP Production in Prokaryotic Cells and Transmission Electron Microscopy (TEM)

To monitor the formation of VLPs by recombinant Pr78^Gag^-His_6_-tag protein in bacterial cells (following induction with IPTG), cells were pelleted, washed with 0.1 M PBS and fixed in Karnovsky’s fixative. Next, cell pellets were stained with 1% osmium tetroxide and subjected to dehydration using graded ethanol solutions. Finally, cell pellets were fixed in epoxy resin (agar 100). Ultrathin sections on 200 mesh copper (Cu) grids were negatively stained with 1% uranyl acetate followed by lead citrate double stain and analyzed using a FEI Tecnai Biotwin Spirit G2 transmission electron microscope.

### *In Vitro* Assembly of Recombinant Pr78^Gag^-His_6_-tag Protein to Form VLPs

To observe *in vitro* assembly of VLPs, purified recombinant Pr78^Gag^-His_6_-tag protein (in 20 mM Tris (pH 7.4) containing 1.0 M NaCl and 10 mM dithiothreitol) was mixed with yeast tRNA at a nucleic acid to protein ratio of 4% (w/w), placed in a Slide-A-Lyzer® 10 KDa dialysis cassette G2 (Thermo Scientific), and dialyzed against 20 mM Tris (pH 7.4) containing 150 mM NaCl and 10 mM dithiothreitol overnight at 4 °C. Following dialysis, ~400 µl was recovered that was concentrated using Amicon® Ultra 15 (30,000 molecular weight cut-off membrane) to a final volume of ~250 µl of which 8–10 µl was spotted on a carbon coated formvar gr id (Proscitech, Australia), air dried, and stained with 1% uranyl acetate for TEM observation.

## Electronic supplementary material

Supplementary Data
