# Supplementary material for: Expression, purification, and characterization of biologically active full-length Mason-Pfizer monkey virus (MPMV) Pr78Gag
Source: Sci Rep. 2018 Aug 7;8:11793. doi: 10.1038/s41598-018-30142-0 (PMC6081465; doi:10.1038/s41598-018-30142-0)
Supplement: Supplementary file 1 — Supplementary Data [file 41598_2018_30142_MOESM1_ESM.pdf]

Supplementary Data

Expression, purification, and characterization of biologically active full-length Mason-Pfizer monkey virus (MPMV) $Pr78^{Gag}$

Fathima Nuzra Nagoor Pitchai[1], Lizna Ali[1], Vineeta N. Pillai[1], Akhil Chameettachal[1], Syed Salman Ashraf[2], Farah Mustafa[3], Roland Marquet[4,*], Tahir A. Rizvi[1,*]

[1]Department of Microbiology & Immunology,
College of Medicine and Health Sciences (CMHS)
United Arab Emirates University (UAEU)
Al Ain, United Arab Emirates (UAE)
[2]Department of Chemistry,
College of Science (COS)
United Arab Emirates University (UAEU)
Al Ain, United Arab Emirates (UAE)
[3]Department of Biochemistry,
College of Medicine and Health Sciences (CMHS)
United Arab Emirates University (UAEU)
Al Ain, United Arab Emirates (UAE)
[4]Université de Strasbourg, CNRS,
Architecture et Réactivité de l'ARN, UPR 9002,
Strasbourg, France

*To whom correspondence should be addressed.

TAR, Department of Microbiology & Immunology, College of Medicine and Health Sciences (CMHS), United Arab Emirates University (UAEU), P.O. Box 17666, Al Ain, UAE.
Tel: (+971) 3-713-7514; Fax: (+971) 3-767-1966; E-mail: tarizvi@uaeu.ac.ae

RM, Université de Strasbourg, CNRS, Architecture et Réactivité de l'ARN, UPR 9002, IBMC, 15 rue René Descartes, 67084 Strasbourg cedex, France.
Tel: (+33) 388 41 70 54. Fax: (+33) 388 60 22 18; E-mail: r.marquet@ibmc-cnrs.unistra.fr

Supplementary Data

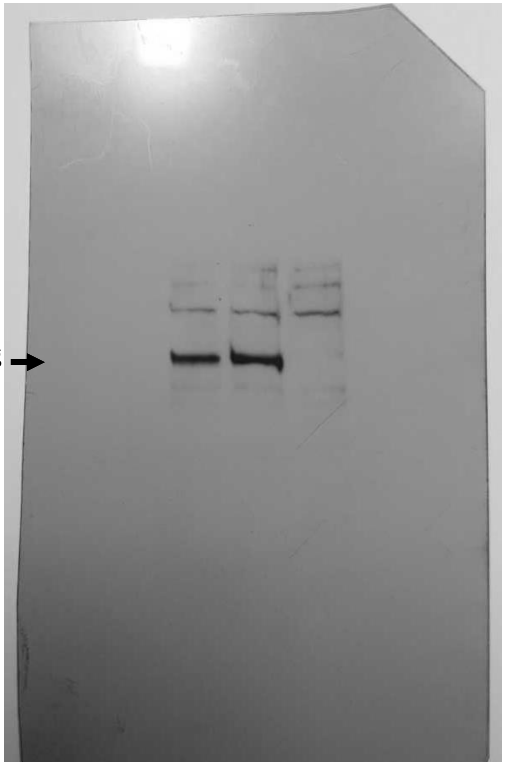

Pr78^{Gag} Expressed in Transfected Cells
Blot used for Figure 8C
Panel I
Image Captured using X-ray film

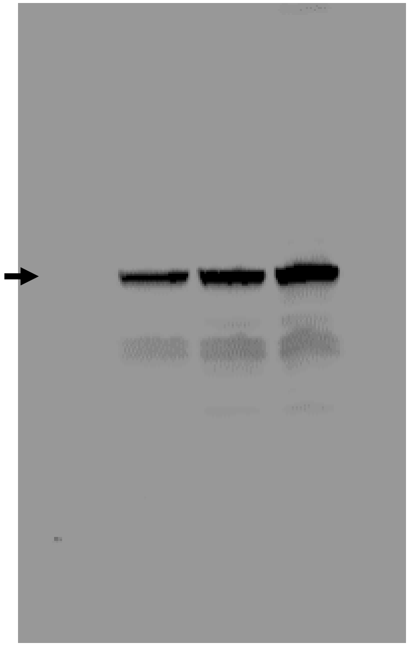

42 kDa β-Actin
Blot used for Figure 8C
Panel II
Image Captured using
Typhoon FLA 9500

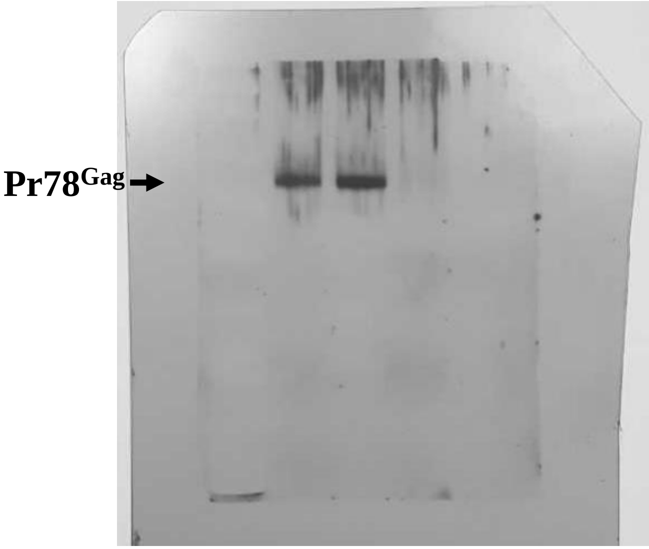

Pr78^{Gag} Viral
Blot used for Figure 8C
Panel III
Image Captured using X-ray film

Supplementary Data

100 bp Ladder
Negative Control
FN7 (+ His)
FN9 (- His)
Mock
Positive Control

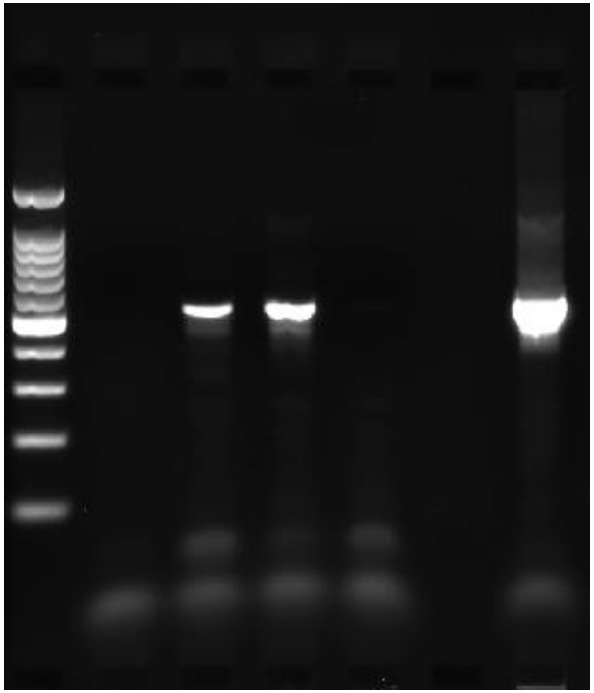

Cytoplasmic RNA (30X)
Agarose Gel used for Figure 8C
Panel IV

100 bp Ladder
Negative Control
FN7 (+ His)
FN9 (- His)
Mock
Positive Control

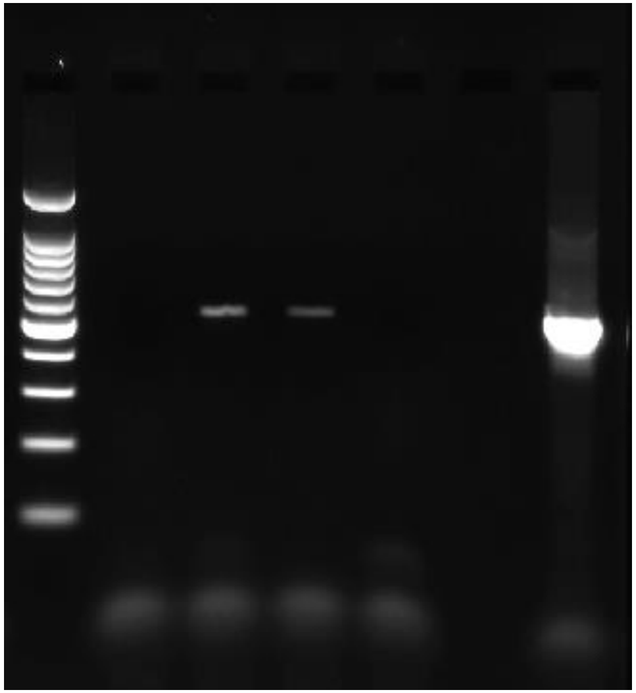

Viral RNA (30X)
Agarose Gel used for Figure 8C
Panel V

100 bp Ladder
Negative Control
FN7 (+ His)
FN9 (- His)
Mock
Positive Control

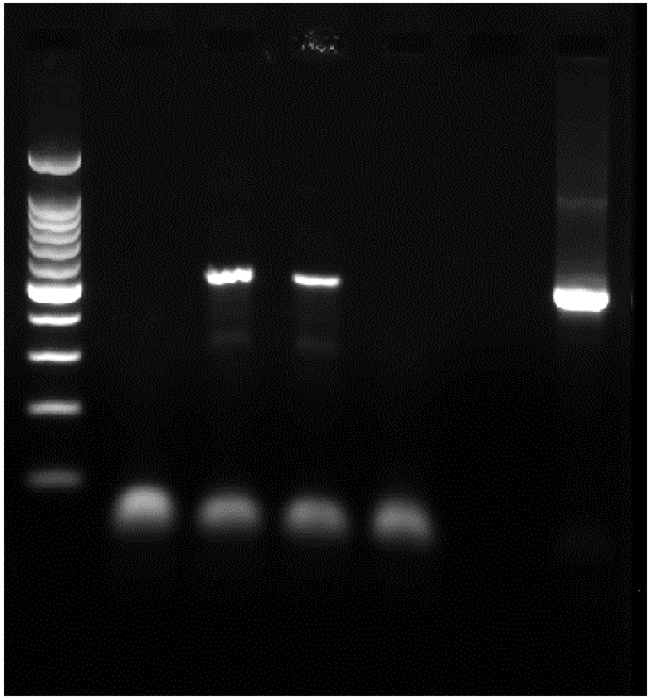

Viral RNA (50X)
Agarose Gel used for Figure 8C
Panel VI

Supplementary Data

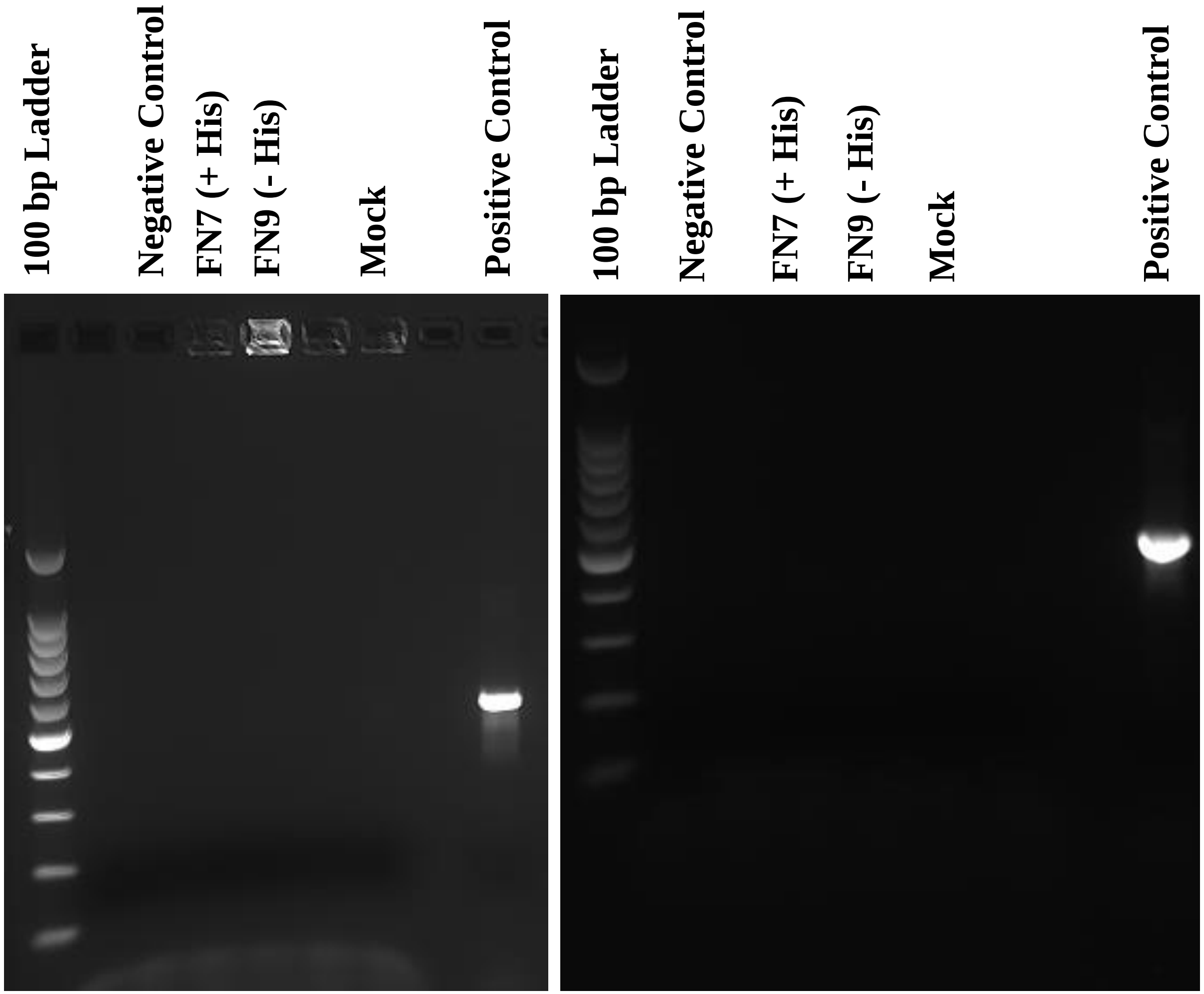

DNase-treated Cytoplasmic RNA (30X)

DNase-treated Viral RNA (30X)
